# Incidence of high chromogranin A serum levels in patients with non metastatic prostate adenocarcinoma

**DOI:** 10.1186/1756-9966-29-166

**Published:** 2010-12-17

**Authors:** Marialuisa Appetecchia, Aurela Meçule, Giuseppe Pasimeni, Concetta V Iannucci, Piero De Carli, Roberto Baldelli, Agnese Barnabei, Giovanni Cigliana, Isabella Sperduti, Michele Gallucci

**Affiliations:** 1 Regina Elena National Cancer Institute, Rome, Italy

## Abstract

**Background:**

ChromograninA in prostate carcinoma (PC) indicate NE differentiation. This tumour is more aggressive and resistant to hormone therapy.

**Patients and methods:**

We analyzed the incidence of pre-operative ChromograninA serum levels in non metastatic PC patients. Serum PSA and ChromograninA were analyzed before treatment. Clinicopathological parameters were evaluated in relation to serum ChromograninA. 486 patients were enrolled.

**Results:**

We found 352 pT2 and 134 pT3. 21 patients were N+. 278 patients had Gleason score levels <7; 173 patients had levels = 7 (122 were 3+4 and 51 4+3); and 35 patients with levels >7. Median PSA pre-operative level was 7.61 ng/ml. PSA was significantly associated with pT stage (pT2 with PSA abnormal 23.6% *vs *pT3 48.5%, p < 0.0001) and with a Gleason score (PSA abnormal 60% in the Gleason score was >7 *vs *29.5% in the Gleason score = 7 *vs *27.3% in the Gleason score <7, p < 0.0001). In 114 patients pre-operative ChromograninA levels were elevated (23.5%). Serum ChromograninA levels had no significant association with PSA (p = 0.44) and pT stage (p = 0.89). abnormal ChromograninA levels increased from a Gleason score of <7 (25.5%) to >7 (31.4%) (p = 0.12). The serum ChromograninA levels in the two groups of patients were subdivided before and after 2005 on the basis of different used assays, showing no correlation with serum ChromograninA and other parameters.

**Conclusions:**

This study showed that ChromograninA levels correlated to NE differentiation and possible aggressiveness of PC. Pre-operative circulating ChromograninA could complement PSA in selecting more aggressive PC cases, particularly in the presence of a higher Gleason score. Complementary information is provided by the absence of a correlation between serum ChromograninA and PSA levels.

## Background

Prostate cancer (PC) has become the most prevalent malignant tumour in men in the Western World and the second leading cause of male cancer-related death. Initially, most tumours present androgen-sensitive carcinomas but the proportion of undifferentiated histology becomes more apparent when correlated to clinical progression and the development of hormone resistance occurrence [1,2]. The explanation of the conversion of a hormone-sensitive status to a hormone-insensitive one is currently one of the most critical areas of debate in prostate carcinoma. Prostate specific antigen (PSA) is at present the better pre-treatment predictor of the disease and of its outcome after treatment. However, its sensitivity and specificity are not yet sufficient to make it the perfect screening test for prostate cancer. Prostate tumour is composed of a heterogeneous population of cells with different levels of androgen dependency. A decline in serum PSA does not always indicate a cure of cancer, as PSA production is androgen dependent and as a result the dedifferentiation of neoplastic cells gradually lose their capacity to produce PSA. Consequently, serum PSA is less reliable as a tumour marker in patients with high tumour grades and in hormonally treated patients with disseminated disease.

Approximately 50% of all prostate carcinomas reveal NE features. NE cells are found in all stages of prostate cancer and are "freely" dispersed throughout the tumour. Independent groups of researchers have shown that NE cells lack or do not express the androgen receptor [3]. NE cells produce specific proteins, such as neuron specific enolase (NSE), chromograninA (CgA), bombesin, serotonin, somatostatin, a thyroid-stimulating-like peptide, parathyroid hormone-related peptides, and calcitonin which are secreted into the blood stream. These NE hormones have growth-factor activities on both normal and malignant prostatic tissues. A number of them have also been shown to activate or be activated by oncogenes, as well as being functionally related to oncogenes [4,5]. NE cells may also have a paracrine impact on the stroma cell growth factor release [4]. It has been hypothesized that the paracrine effect of the neurosecretory cell products on adjacent cells can contribute to the growth and differentiation of prostatic cells. In fact, stromal growth factors, such as epithelial growth factor (EGF), insulin-like growth factor (IGF), fibroblast growth factor (FGF) balance changes may be responsible for the progression of prostate cancer too [6]. Thirteen years ago, Kadmon et al. reported that circulating CgA, main NE product, was elevated in 48% of subjects with metastatic prostate cancer [7]. This evidence highlighted the importance of serum CgA monitoring in prostate cancer patients [7]. ChromograninA is an excellent marker of NE cells and of neuroendocrine differentiation (NED) in prostate carcinomas either in terms of tissue or the blood stream [3]. The detection of this marker in the blood of patients with prostate cancer indicates a NED, either of a primary tumour or an association with a metastases [8]. Tumours displaying NE features are reported to be more aggressive and resistant to hormone therapy [9]. Some authors claimed that CgA is an independent prognostic marker in clinical under-staging of PC [10], while others failed to find this correlation [11]. Many groups have attempted to identify risk factors that could help to early detect more aggressive PC such as those with NE characteristics. The knowledge of such risk factors could facilitate the clinical management of such tumours and prolong survival.

The aim of our study was to analyzed the incidence of pre-operative circulating CgA in a population of non metastatic prostate cancer patients. Serum PSA levels, pathological staging and the Gleason score were also evaluated.

## Methods

This is a single centre study.

The present retrospective study examined data of 740 consecutive patients with clinically non-metastatic prostate adenocarcinoma that were enrolled from 2003 to 2006 at the Urology Department of our Institute for radical prostatectomy (RRP).

Inclusion criteria considered were:

• No previous hormonal or radiation therapy

• No previous surgery on the prostate gland

• Histologically proven adenocarcinoma of the prostate at biopsy and confirmed at RRP.

• No positive surgical margins.

• One hundred ten (14.9%) patients were excluded from the study for missing data.

• One hundred forty four (19.5%) patients were not considered as they were submitted to neoadjuvant hormonal therapy.

A total of 486 patients were included in the present analysis and were evaluated for all the variables considered (pathologic tumour stage, tumour grade, serum total PSA and CgA, age).

None of these patients had previous or concomitant history of other malignant disease, adrenal incidentalomas, hepatic and/or renal impairment and/or uncontrolled blood hypertension.

Similarly, none of the patients were taking drugs known to alter the metabolism and secretion of CgA, such as nitrates and proton pump inhibitors.

An informed consent form was obtained from all patients for all the procedures carried out. The investigation was approved by the local ethical committee.

All patients had a biopsy clinically proven T2-T3 N0 M0 prostate adenocarcinoma, as determined by digital rectal examination, transrectal ultrasonography, bone scan, and computed tomography (CT).

All patients were submitted to RRP.

All RRP specimens were evaluated at our Institute according to routine procedure by the same expert uropathologist.

In all patients the tumour stage was assigned according to the 2002 TNM classification [12].

The tumour grade was described at RRP according to the Gleason score grading system [13].

Blood specimens were obtained in all patients in the early morning, after an overnight fast.

In all patients a blood sample was collected in the early morning, after an overnight fast for the determination of serum total PSA and CgA. All samples were obtained at least 3 weeks after any prostate manipulation before the surgical procedure.

Blood for serum total PSA and CgA assessments was collected in a frozen vial until plasma separation.

All serum and plasma samples were immediately frozen and stored at -20 C until analysis.

ChromograninA was measured with the enzyme-linked immunoabsorbent assay (ELISA-DakoCytomation, Italy) until April 2005 and with the immunoradiometric assay (CGA-RIACT, CIS BIO INTERNATIONAL-France) thereafter.

Chromogranin A ELISA Kit is designed for the quantitative determination of CgA in human plasma (EDTA or heparin). The kit can be used for measuring CgA in the 10 to 500 U/L range. The ELISA kit is a double antibody sandwich assay where samples and conjugates are incubated simultaneously in antibody-coated wells. The imprecision of the assay is less than 9% over the whole measuring range.

CGA-RIACT is a solid-phase two site immunoradiometric assay. Two monoclonal antibodies were prepared against sterically remote sites on the CGA molecule. The first one was coated on the solid phase (coated tube), while the second one, was radio-labelled with iodine 125, and used as a tracer.

CGA (molecules or fragments) present in the standard or samples to be tested were "sandwiched" between the two antibodies. Following the formation of the coated antibody/antigen/iodinated antibody sandwich, the unbound tracer was easily removed by washing it. The radioactivity bound to the tube was in proportion to the concentration of CGA present in the sample.

Reference serum values of 95% of 162 presumed normal individuals were between 19.4 and 98.1 ng/ml, with the median at 41.6 ng/ml. The detection limit of this kit was 1.5 ng/ml. The inter-assay and the intra-assay coefficient of variation of CgA assay was 5.8% and 3.8%, respectively. The normal reference value reported by the kit for CgA was <98.1 ng/ml.

The reference upper value of CgA for the two assays was 20 U/L and 90 ng/ml, respectively.

For each patient, the same serum sample was also used to determine total PSA levels (Total PSA Elecsys-Roche).

All samples were evaluated in the laboratory of the Clinical Pathology Laboratory at our Institute.

After RRP, patients were all followed with PSA determination (monthly during the first year and thereafter every 3 months), bone scan (yearly), CT or MNR (yearly or at PSA progression).

According to literature [14], biochemical PSA progression was defined as the first occurrence of a PSA increase over 0.2 ng/ml, with a value confirmed at two consecutive determinations with a two week interval.

### Statistical analysis

For the statistical analysis, patients were classified on the basis of the pathological T stage in pT2 and pT3 patients (no pT4 was found and only 21 patients showed N+ disease).

On the basis of RRP, Gleason score patients were classified in a Gleason score of <7, Gleason score = 7 and >7. ChromograninA values were standardized in order to obtain homogeneous data for the statistical evaluation.

Based on the pre-operative serum PSA levels and previous experience in literature [15], our patients were subdivided in ≤10.0 ng/ml and >10.0 ng/ml.

Descriptive statistics (median, mean, range, standard deviation) were used to characterize the population. Categorical variables were assessed by the Pearson Chi-square test. Student's t-test was used to compare mean values. Spearman correlation coefficients were calculated to measure the association among CgA and other parameters. A p value ≤ 0.05 was considered statistically significant.

All statistical analyses were performed by the SS version 13.0

## Results

The clinical and pathological characteristics of our population are described in Table 1.

**Table 1:** Clinical and pathological characteristics of PC patients

Number of cases	486
Age (yr)	

Median	64 (range 44-75)

Preoperative Serum PSA (ng/ml)	

Median	7,61 (range 0,75-125)

Preoperative serum PSA ≤10 ng/ml	

Number of cases	148 (30.5%)

Preoperative serum PSA >10 ng/ml	

Number of cases	338 (69.5%)

Preoperative Serum CgA (U/L)	

Number of cases	216

Mean value	25.24 ± 39.21(range 2-340)

Median value	14

Cg A > 20 U/L	64

Preoperative Serum CgA (ng/ml)	

Number of cases	270

Mean value	79.26 ± 100.50 (range 12-1064)

Median value	55

Cg A > 90 ng/ml	50

Pathological stage	

T stage	

pT2	352 (72.4%)

pT3	134 (27.6%)

N Stage	

pN+	21 (4.3%)

Histological Gleason score < 7	278 (57.2%)

Histological Gleason score = 7	173 (35.6%)

Histological Gleason score >7	35 (7.2%)

The present study included 486 patients (median age 64 yrs, ranging from 44-75).

The TNM classification staging were found to be 352 pT2 (72.4%) and 134 pT3 (27.6%).

Twenty one patients (4.3%) showed regional lymph node disease (N+).

The histology tests examined found 278 tissues with a Gleason score of <7 (57.2%); 173 with a Gleason score = 7 (35.6%), of these 122 had a score of 3+4 (705% and 51 with a 4+3 (29.5%) and 35 with a Gleason score of >7 (7.2%).

The median PSA circulating pre-operative level was 7.61 ng/ml (range 0.75-125).

One hundred forty eight patients (30.5%) had a pre-operative PSA ≤10 ng/ml; 338 patients (69.5%) had a PSA > 10 ng/ml.

PSA was significantly associated with pT stage (pT2 with PSA abnormal 23.6% *vs *pT3 48.5%, p < 0.0001) and Gleason score (PSA abnormal 60% in the Gleason score >7 *vs *29.5% in the Gleason score = 7 vs 27.3% in the Gleason score <7, p < 0.0001).

In 114 patients pre-operative circulating CgA levels were elevated (23.5%).

The serum CgA levels had no significant association with PSA (p = 0.44) and pT stage (p = 0.89).

Classifying cases on the basis of the Gleason score (> 7 *vs *= 7 *vs *< 7), abnormal CgA levels increased from a Gleason score of <7 (25.5%) to a Gleason score of >7 (31.4%) (p = 0.12).

In addition, the statistical analysis of serum CgA levels, were carried out separately in the two groups of patients and were then subdivided before and after 2005 (on the basis of a different used assay), showing no correlation among serum CgA and other parameters.

## Discussion

Neuroendocrine (NE) differentiation frequently occurs in common prostate malignancies and it is attracting increasing attention in prostate cancer research. Virtually all prostate adenocarcinomas show NE differentiation as defined by the NE marker chromograninA. Angelsen et al. reported that CgA positive tumours presenting high serum CgA levels, suggested that the CgA should be a useful marker for predicting the extent of NED in prostate cancer [16]. NE differentiation, however, occurs only in the G0 phase of the cell cycle when tumour cells are usually resistant to cytotoxic drugs and radiotherapy. Even NE tumour cells do not proliferate, they produce NE growth factors with mitogenic activity that promote cell proliferation and induce anti-apoptotic features in non-NE cells in close proximity to NE cells through a paracrine mechanism [17]. Neoplastic epithelial cells may become more responsive to NE products by upregulation of the neuropeptides receptors, or may stimulate NE cells to up-regulate the secretion and synthesis of their products [4]. Neuroendocrine tumour cells lack androgen receptors and are androgen insensitive in all stages of the disease. Even though androgen depletion results in apoptosis of the epithelial cells, it seems that it is not able to eliminate all cancer cells, and over time, an androgen-independent NE population emerges and ultimately predominates [4]. However some authors disagree with this finding [18]. Prostate cancer cells with NE features escape programmed cell death [19]. Even under androgen deprivation, only 0.16% of NE tumour cells show apoptotic activity. This indicates that NE tumour cells represent an immortal pattern in prostate cancer. PSA is an important tool for detecting prostate cancer. However, it was reported that the diagnostic role of serum PSA in assessing the treatment efficacy in patients with hormone-refractory disease did not correlate with changes in pain symptomatology and disease outcome [20]. Some authors reported that high levels of CgA allowed prognostic information independently from PSA [21], while others failed to show the same results [6,10,11,22,23]. Neuroendocrine differentiation also appeared to be associated with the androgen-refractory state and a poor prognosis [6,23-26]. It was reported that prostate cancer with a significant NE component is common in the advanced stage of the disease, especially in those patients who do not have elevated serum PSA levels [7,25,27,28], but its diagnostic role in non metastatic disease is still a matter of debate [8,29,30]. We analyzed serum CgA levels in patients who were diagnosed with a prostate cancer before surgery. In our population 23.5% of all patients showed elevated pre-treatment circulating CgA levels. It is worthy to note that our population showed pre-treatment supra-normal CgA serum levels in the absence of distant metastases. In our series of patients serum CgA levels had no significant association with PSA.

According to other authors [25,31], we found that CgA depicted a significant trend in association with high-grade disease. We did not observe any associations in our assessment of pathological stages.

## Conclusions

According to our study, ChromograninA levels demonstrated a correlation with NE differentiation and possible aggressiveness of PC. This finding suggests that pre-operative circulating CgA determination could have a potential role in the clinical management of PC patients and could complement the PSA assay in an early selection of more aggressive PC such as those with NE features, particularly in those patients showing a higher Gleason score.

## Competing interests

The authors declare that they have no competing interests.

## Authors' contributions

MA made substantial contributions to the conception, design and coordination of the study as well as the preparation of the final version of the manuscript.

AM, GP and CVI were involved in the process of patient selection and in the data collection.

PDC was responsible for enrolling patients.

RB and AB participated in data collection.

GC performed the tests in the laboratory.

IS carried out the data analyses.

MG participated in the coordination of the final version of the manuscript. All authors have read and approved the final manuscript.
